# Inactivation of *Salmonella* and avian pathogens on hatchery eggs using gas phase hydroxyl-radical process vs formaldehyde fumigation: Efficacy, hatching performance and grow-out of Chickens

**DOI:** 10.1016/j.psj.2025.105023

**Published:** 2025-03-10

**Authors:** Harleen Kaur Dhillon, Mahdiyeh Hasani, Brenda Zai, Kathryn Yip, Lara Jane Warriner, Ivy Mutai, Belinda Wang, Michael Clark, Sudhakar Bhandare, Keith Warriner

**Affiliations:** a Department of Food Science, University of Guelph, Guelph, ON, Canada; b Department of Population Medicine, University of Guelph, Guelph, ON Canada; c School of Veterinary Medicine and Science, University of Nottingham, Leicestershire, UK

**Keywords:** Eggs, Hatchery, Disinfection, *Salmonella*, Hydroxyl-radical

## Abstract

Hatcheries have been identified as a significant source of *Salmonella* within poultry production. Consequently, there is a need for effective egg disinfection methods that can reduce the pathogen burden while preserving the egg integrity and embryo. The metrics for a successful egg disinfection method are typically a reduction in Total Aerobic Count (TAC) while retaining hatching rates. In this study, a gas phase hydroxyl-radical process was validated and verified as a hatchery egg disinfection method. The process is based on applying a hydrogen peroxide mist in combination with ozone gas and UV-C to generate antimicrobial hydroxyl radicals. The treatment (2 % hydrogen peroxide, 20 ppm ozone and 19 mJ/cm^2^ UV-C; designated as HR) for inactivating *Salmonella* (serotypes Enteritidis and Typhimurium) inoculated onto eggs could eliminate the pathogen (>5 log CFU/egg reduction) but left residual TAC (1.53 log CFU/egg reduction). Surface sterilization was achieved by a pre-treatment of eggs with the photo-catalyst riboflavin (13.75 mM) followed by 3 % hydrogen peroxide delivered at 70 °C prior to the hydroxyl-radical treatment (3 % hydrogen peroxide, 20 ppm ozone and 114 mJ/cm^2^ designated HRS). The surface sterilization of eggs coincided with the removal of the cuticle layer with the HRS treatment but not HR. The cuticle layer was also compromised by formaldehyde treatment. When the different treatments were applied to fertile hatchery eggs (n=50 eggs per treatment group), there was no significant difference in hatchery rate (64-74 %), with hatch to fertility being higher for disinfected eggs (89-97 %) compared to the non-treated control (80 %). The seven-day mortality (0 – 2 birds) and feed conversion ratio (1.59 – 1.75 kg/kg feed) did not significantly differ between the treated vs controls. The HR treatment could eliminate *Enterococcus faecium, Escherichia coli* (>5 log CFU/egg reduction) although HRS was required to inactivate *Pseudomonas aeruginosa* (>5 log CFU/egg reduction) and reduce *Aspergillus niger* spores (3.08±2.25 log CFU reduction). The study has provided treatment options for hatchery egg disinfection and alternative to formaldehyde treatment.

## Introduction

Hatchery eggs have been identified as a significant source of *Salmonella* whereby the pathogen can become associated with the developing embryo then disseminated through the flock when the birds are raised in broiler or hatchery egg production (Sivaramalingam, *et al*., 2013). Once established within a flock, then eggs and poultry meat can carry *Salmonella* through to the end-consumer leading to cases or outbreaks of salmonellosis (Wang, *et al*., 2023). Consequently, there is a need to implement controls as a preventative approach as part of an overall *Salmonella* control program. In relation to hatchery egg disinfection, there are operations that apply no treatment to ensure the cuticle is protected (Melo, et al., 2019). The cuticle is an outer protective layer derived from the mucus coating of freshly laid eggs that is readily removed by physical abrasion and/or washing (Leleu, *et al*., 2011). Those hatcheries that decontaminate eggs typically apply formaldehyde gas generated from the reaction of formalin with potassium permanganate within a sealed chamber (Cadirci, 2009; Fasenko, *et al*., 2009). Formaldehyde is hazardous to workers and generates toxic byproducts that have led to alternative egg disinfection methods being sort (Melo, *et al*., 2019). Alternative treatments to formaldehyde have included electrolyzed water, chlorine dioxide hydrogen peroxide, and ultraviolet light (Coulibaly, *et al.*, 2024; Cox, *et al*., 2002; Keita, *et al*., 2016; Melo, *et al*., 2019; Turtoi and Borda, 2014; Wells, *et al*., 2010). Such methods have a limited decontamination efficacy with regards to microbial reduction or negative effect on hatchery rates (Keita, *et al*., 2016).

An alternative egg disinfection method based on the gas phase hydroxyl-radical process has shown the potential to inactivate *Salmonella* inoculated onto hatchery eggs while preserving the cuticle layer and maintaining hatchery rates (Zai *et al*., 2023). The treatment is based on the UV-C (at 254 nm) degradation of hydrogen peroxide vapor and ozone gas to produce antimicrobial hydroxyl-radicals (Warriner *et al*., 2021). In relation to egg disinfection, the hydroxyl-radical process based on 2 % v/v hydrogen peroxide delivered as a mist and 20 ppm ozone delivered within a continuous reactor containing UV-C lamps delivering a dose of 19 mJ/cm^2^ supported a >5 log CFU/egg reduction of *Salmonella enterica* subsp *enterica* Enteritidis (Zai, *et al*., 2023). There was no negative effect on the integrity of the cuticle layer, Haugh Units, or the hatchery rate of eggs compared to non-treated controls. In the current study, the range of *Salmonella* strains was extended and avian pathogens included. Specifically, avian *E. coli* is linked to colibacillosis that causes significant economic losses in the poultry sector (Kwon, *et al*., 2013). In a similar manner, *Pseudomonas aeruginosa* is a potent avian pathogen that causes respiratory infections exhibited as omphalitis (Walker, *et al*., 2002). *Enterococcus faecalis* is an opportunistic avian pathogen that leads to reduced hatchability rates (Reynolds and Loy, 2020). *Aspergillus* mold is a further avian pathogen that results in mortality of developing embryos thereby leading to a decreased hatch yield with associated economic losses (Williams, *et al*., 2000).

A further part of the study was to enhance the disinfection efficacy of the hydroxyl-radical process through inclusion of a photo-sensitizer to support surface sterilization of egg surfaces. Specifically, the inclusion of riboflavin (vitamin B2) as the photo-sensitizing agent has previously been applied to enhance the antimicrobial action of ultraviolet and Blue light (400-500 nm) through a free-radical propagation mechanism (Liang, *et al*., 2013; Sel, *et al*., 2014). In a related example, when riboflavin is combined with UV-C and hydrogen peroxide there is an increase in the hydroxyl-radical propagation that degraded the stable herbicide, manuron (Chan and Chu, 2009). The authors reported that using the combination of UV-C, hydrogen peroxide (1.4 mM) and riboflavin (0.22 mM) supported a 79 % greater reduction of the herbicide compared to UV-C and hydrogen peroxide alone (Chan and Chu, 2009).

## Materials and methods

The study was subdivided into two parts, with the first developing the hydroxyl-radical process to inactive *Salmonella*, avian pathogens, and total aerobic count of eggshell surfaces. The second part undertook an animal trial to evaluate if the hydroxyl-radical-based treatments affected embryo and chick development in comparison to the current formaldehyde egg disinfection method.

### Microbes and cultivation conditions

The *Salmonella* serotypes tested were Enteritidis BO-1328, P125592, PT8, P125088, S1342, and Typhimurium 3360, 345/66, C5, SL 1027 that were isolated from the hatchery environment or raw egg and were donated by the University of Nottingham, UK and Public Health Agency of Canada. *Escherichia coli* K12, *Enterococcus faecalis* 19433, and *Pseudomonas aeruginosa* 15442 were obtained from ATCC (Atlanta, GA, USA). The bacteria were maintained at -80°C in Tryptic Soy Broth (TSB; Thermo Fisher, Mississauga, ON, Canada) containing 70 % glycerol (Sigma-Aldrich, Whitby, ON, Canada), then revived by streaking on Tryptic Soy Agar (TSA; Thermo Fisher) and incubating at 37 °C for 24 h. Cultures were prepared by inoculating 50 ml of TSB with the individual strains and incubating overnight at 37 °C. The cells were harvested by centrifugation (5000xg 10 min; Avanti J-20 XPI centrifuge; Beckman Coulter, Mississauga, ON, Canada). The supernatant was decanted, and cell pellet resuspended in saline to a final optical density at 600 nm of 0.2 that equated to approximately 8 log CFU/ml. The cells suspensions were held at 4°C until required.

*Aspergillus niger* NU 320 spores were prepared by inoculating Potato Dextrose Agar (PDA; Thermo Fisher) that was subsequently incubated at 25 °C for 7 days. The spores were harvested by flooding the plate with sterile distilled water and scraping the growth using a sterile hockey stick. The spore suspension was harvested by centrifugation (5000xg 10 min) and washed once with sterile distilled water. The spore pellet was resuspended in sterile distilled water, and the spore count was determined by plating onto PDA that was subsequently incubated at 25°C for 5 days. The spore suspension was held at 4°C until required.

### Egg inoculation

Unwashed, non-fertilized hatchery eggs free of cracks and visible debris were obtained from the University of Guelph Poultry Research Station (Arkell, Ontario, Canada). The eggs were stored at 4°C and equilibrated at 25°C for 1 h to remove residual condensate. The eggs were placed on an egg tray and then spot inoculated at different areas of the eggshell with 10×10 µl of an 8 log CFU/ml of the test strain to ensure a defined cell loading. The inoculated eggs were held at room temperature for 30 min to allow cells/spores to attach.

### Gas-phase hydroxyl-radical process

The gas phase hydroxyl-radical reactor has previously described by Zai *et al*. (2023). In brief, the unit was constructed from a stainless-steel frame that housed ten UV-C lamps (23 W; 254 nm) that were positioned over a motorized conveyor belt system. The UV-C intensity was measured at four different points on the belt surface using a radiometer (Trojan Technologies Inc., London, ON, Canada) and found to have an average output of 3.8 mW/cm^2^. Within the reactor, the hydrogen peroxide (2%) was delivered as a mist at a flow rate of 30 ml/min. Ozone gas was generated by two 12 W UV lamps emitting at 184 nm positioned on either side of the conveyer. Air was flowed over the lamps via a pump and introduced into the chamber via vents. The concentration of ozone measured in the absence of UV-C lights or hydrogen peroxide spray was via an internal feedback system that was set at 20 ppm (Zai et al., 2023). The temperature of the chamber was maintained at 29 °C via hot air generated by a heating block located at the top of the unit.

For the hydroxyl-radical treatment (HR), the inoculated eggs (n = 5 per treatment) were placed on the tray and then passed through the reactor with a 10s treatment time and UV-C dose of 19 mJ/cm^2^. The HRS treatment consisted of spraying (3 ml/egg) of a riboflavin (Sigma-Adrich) solution (13.75 – 55 mM) followed by a further spray (3 ml/egg) of pre-warmed (48 -70 °C) hydrogen peroxide solution. The eggs were then passed through the gas phase hydroxyl-radical reactor with a 30 s transit time (equate to a UV-C dose of 114 mJ/cm^2^).

### Formaldehyde treatment

Formaldehyde treatment was performed within a 3.5 liter jar with 3 eggs being treated each time (total of 5 eggs per treatment). In a fume hood, a glass dish holding 1.2 ml formalin (10%) was activated by the addition of 0.6 g of potassium permanganate. The lid of the jar was then secured and sealed with the eggs being treated for 20 mins (Cadirci, 2009).

### Recovering and enumerating survivors from eggs

The non-treated (controls) and treated eggs were individually placed in a plastic pouch containing 50 ml TSB supplemented with 20% w/v glycerol, as described by Zai *et al*. (2023). The egg was manually massaged for 2 min and then held at 25°C for 1h to enable cells to transition from the dry to hydrated environment. A dilution series was prepared from the solution that was subsequently plated onto the appropriate agar. For *E. coli* K12, *E. coli* /Coliform Petri Films (Neogen, Edmonton, AB, Canada) incubated at 37 °C for 24 h was applied. The rinse from the egg was combined with an equal volume of double-strength lactose broth that was enriched at 37°C for 24 h. Enriched samples positive for growth were streaked onto MacConkey agar (MAC, Thermo Fisher) that was subsequently incubated at 37 °C for 24 h. Presumptive positive colonies were confirmed as *E. coli* using the IMViC tests.

*E. faecalis* was enumerated on MacConkey #2 agar (MAC2; Thermo Fisher) incubated at 37 °C for 48 h. The egg rinse solution was then added to an equal volume of TSB and then enriched for 24 h at 37 °C. The enriched sample was then streaked onto MAC2 agar plates that were incubated at 37 °C for 48 h and then examined for typical colonies (small red-colored colonies). *P. aeruginosa* was enumerated on *Pseudomonas* Agar (Thermo Fisher) incubated at 37 °C for 24 h. The rinse was enriched at 37 °C for 24 h in TSB and then streaked onto *Pseudomonas* agar that was incubated at 37 °C for 24 h and then inspected for typical colonies with blue pigmentation.

*Salmonella* was enumerated on Xylose Lysine Deoxycholate agar (XLD, Thermo-Fisher) that was incubated at 37 °C for 24 h. As with *Enterobacter* and *Pseudomonas*, the egg rinse was added to an equal volume of TSB and then enriched for 24 h at 37 °C. Aliquots (0.1 ml) of the enriched culture were spotted onto Modified Semi-solid Rappaport Vassiliadis medium (MSRV; Thermo Fisher) that was incubated overnight at 42 °C. Presumptive positive colonies were streaked onto XLD agar and incubated at 37 °C for 24 h with confirmation testing being performed using isothermal PCR Molecular Detection System (3M, London, ON, Canada). *Aspergillus niger* was enumerated on PDA incubated for 5 days at 25 °C.

The Total Aerobic Count and Yeast & Mould count was performed on non-inoculated eggs (n = 5 per treatment). Here the eggs were pre-incubated in TSB containing 20 % glycerol. A dilution series was prepared with the TAC being enumerated on Plate Count Agar (PCA, Thermo Fisher) at 34 °C for 48 h. Yeasts & Moulds were determined by plating the dilution series on PDA that was incubated at 25 °C for 5 days. In parallel, the egg in TSB was enriched by incubating at 34 °C for 48 h, and growth or non-growth was recorded.

### Egg quality assessment

Cuticle staining was performed using the Cuticle Blue staining technique (Roderiguez-Navarro *et al*., 2013). Here, the eggshell color was measured at five different parts of the egg using a colorimeter (Konica Minolta Chrome Meter CR-400, Konics Minorta Sensing Americas Inc, NJ, USA). The eggs (n = 30 per treatment) were submerged in 1% Cuticle Blue stain (MS Technologies Ltd, Northamptonshire, UK) for 5 min followed by a rinse in distilled water. The egg was then allowed to air dry for 1 h before re-taking the color measurements that were then used to determine the change in color (ΔEab) (Zai *et al*., 2023).

The Haugh Unit of eggs (n = 30 per treatment) was determined using an Egg Analyzer (ORKA Food Technology, Utah, USA). Unfertilized eggs (control and treated) were stored within cartons for 35 days at 4 °C then analyzed using the egg analyzer to assess the egg weight, albumin height, and yolk color that was subsequently used to calculate the Haugh Unit (Zai *et al*., 2023).

### Animal trials

All animal procedures conducted in this study adhered to the guidelines established by the Canadian Council on Animal Care and received approval from the University of Guelph Animal Care Committee (AUP #4565).

Eggs from Ross 708 (51-week-old), free from physical defects or cracks, were obtained from the Arkell Research Station-Poultry Unit in Guelph, Ontario, Canada, within 48 h of laying (n = 200), divided into 4 groups, including non-treated control. The treatments applied were the HR as described by Zai *et al*. (2023). Specifically, 2 %v/v hydrogen peroxide at a flow rate of 40 ml/min with 20 ppm ozone and a UV-C dose of 19 mJ/cm^2^. The HRS treatment involved initially spraying the eggs with riboflavin (13.75 mM) followed by 3 %v/v hydrogen peroxide delivered at 70 °C before passing through the hydroxyl-radical reactor operating at 3 %v/v hydrogen peroxide, 20 ppm ozone and a UV-C dose of 114 mJ/cm^2^. The formaldehyde treatment was performed within a sealable jar with the antimicrobial gas being formed from the addition of permanganate to 1.2 ml of formalin. The eggs were treated for 20 min within the sealed jar before being removed.

The control and treated eggs were returned to the hatchery and stored at 14–16 °C with 80 % relative humidity for 2 days before being transferred to a Nature Form I Series setter maintained at 37.5 °C and relative humidity of 66 %. After 18 days, candling was performed to assess the embryo development, with those showing an absence of embryo being discarded. The remaining eggs were transferred to a Nature Form I Series hatcher (37 °C, 55 % relative humidity). On Day 21 of the incubation, samples were removed from the hatcher approximately two hours post-hatch. The hatchery rate was calculated based on the total number of chicks from the original number of eggs. Hatch of Fertility (HOF) was based on the total number of chicks and on the percentage of eggs showing viable embryos after candling that went onto hatch. The resultant chicks were checked for unhealed navels and red hocks. The chicks were taken from the treatment group and separated into sub-groups of five birds.

The broilers were housed in Ford Dickison Inc. pullet/ cockerel rearing cages. Temperature and light controls were programmed for 40 days according to industry standards (Arkell personal communication). The initial room temperature was set to 32 °C and was eventually lowered to 20 °C by the end of the observation period. The lighting program began at an intensity of over 20 lux with a 22L:2D cycle and was gradually reduced to 10 lux with a 17 Light :7 Dark cycle by the fourth day.

The feed was weighed and provided to the developing birds within feed receptacles for them to consume ad libitum. Drinking water was provided via automated lubing water nipples, and waste was collected by an under-cage conveyor. The birds were inspected twice daily for general health and behavior along with mortalities being recorded. The weight of birds and feed consumed were performed on Days 7, 14, 21, 28, 35, and 40.

### Statistical analysis

Laboratory trials are duplicate experiments, with five eggs being used for each microbiological challenge experiment. The microbiological data counts were transformed into Log_10_. Thirty eggs per treatment were performed to assess the quality metrics (cuticle staining, Haugh Unit). All data from laboratory trials was analyzed using ANOVA in conjunction with Tukey's Test for multiple comparisons (SAS®, version 9.4; SAS Institute Inc. Cary, NC, USA).

The animal trial data was statistically analyzed using Generalized Linear Mixed Models (GLIMMIX) in SAS®. The assumptions of equal variance and normality for ANOVA were satisfied, and means were compared using the LSD post hoc test. Datasets from the chick grow-out trial were analyzed using a randomized complete block design (RCBD), with cages as the experimental unit. Cages within the room were included in the covariance structure as random effects, with day as a repeated measure. To meet ANOVA assumptions, a lognormal distribution was assumed for the weight variable. Embryo viability and hatchability were analyzed using non-parametric χ² tests. Statistical significance for all datasets was determined at P < 0.05, with a significance level of α set at 0.05.

## Results

### Optimization of hydroxyl-radical process for egg disinfection

Several treatment combinations were assessed for surface sterilizing eggs using the hydroxyl-radical process with the reduction in the TAC as a metric (Table 1). The process variables were the concentration and temperature at which the riboflavin and hydrogen peroxide that was deposited on eggs via an electrospray with ozone and UV-C dose being of fixed value. It was found that there was a significant decrease in TAC by applying the hydroxyl-radical process compared to non-treated controls. The reduction in TAC was independent of the hydrogen peroxide concentration within the range of 1-3 % v/v. The antimicrobial action could be enhanced through delivering the hydrogen peroxide at 70 °C although residual populations were encountered (Table 1). The additional step of a riboflavin pre-spray prior to hydrogen peroxide and passage through the reactor had a significant (P<0.05) TAC reduction although this was dependent on the concentration and applied temperature of the hydrogen peroxide. Specifically, when riboflavin was applied at a concentration of 55 mM concentration, there was a positive correlation between the hydrogen peroxide concentration and log count reduction in TAC, although residual populations of bacteria persisted (Table 1). At lower concentrations of riboflavin there was no significant difference (P>0.05) between hydroxyl-radial treatments with or without the vitamin photo-sensitizer (Table 1). However, when the spray temperature of the hydrogen peroxide was increased to 70 °C a synergistic effect was noted with no bacteria begin recovered from eggs with treatments that applied 13.75 mM riboflavin and 3 %v/v hydrogen peroxide prior to entering the hydroxyl-radical reactor. In comparison, treatments using higher riboflavin concentration gave sporadic positive samples by enrichment (Table 1). Therefore, the treatment taken forward for surface sterilization of eggs was an initial spray with 13.75 mM riboflavin, 3 %v/v hydrogen peroxide delivered at 70 °C followed by passage through the hydroxyl-radical reactor (114 mJ/cm^2^) and 20 ppm ozone. The treatment was designated at Hydroxyl-radical Sterlization (HRS).

**Table 1:** Reduction of total aerobic count (TAC) of shelled eggs treated with different combinations of hydroxyl-radical treatment. Ungraded eggs were separated into groups of six, and the treated hydroxyl radical unit operated with different hydrogen peroxide concentrations. Hydrogen peroxide adjusted to 70°C was applied manually before entering the hydroxyl-radical reactor. Riboflavin was sprayed manually before being sprayed with hydrogen peroxide. The control and treated eggs were transferred to TSB to prepare a dilution series and enriched in parallel by incubating at 34°C.

Treatment	H_2_O_2_ (%)	Temperature (°C)	Riboflavin (mM)	Log CFU/Egg (#Positive/Total Tested)
Control				4.65±0.12a
1	1	48	0	3.17±0.55b
2	2	48	0	3.12±0.18b
3	3	48	0	3.26±0.16b
4	1	48	55	4.33±0.13a
5	2	48	55	3.48±0.54b
6	3	48	55	2.87±0.03c
7	1	48	27.5	3.35±0.22b
8	2	48	27.5	2.68±0.22c
9	3	48	27.5	3.15±0.43b
10	1	48	13.75	2.99±0.65bc
11	2	48	13.75	3.41±0.72b
12	3	48	13.75	3.33±0.43b
13	3	70	0	1.80±1.56d (2/6)
14	3	70	13.75	<1.00e (0/6)
15	3	70	27.5	<1.00d (1/6)
16	3	70	55	<1.00d (1/6)

Means followed by the same letter are not significantly different (P>0.05)

### Inactivation of *Salmonella* and avian pathogens inoculated onto eggs

A comparison was made with respect to the hydroxyl-radical process using a combination of hydrogen peroxide, ozone and UV-C (i.e., HR which would support *Salmonella* inactivation) vs the treatment with the inclusion of photosensitizer to support surface sterilization (HRS). Both the HR and HRS treatments could inactivate *E. faecalis, E. coli and Salmonella* (Table 2). *P. aeruginosa* was reduced by 4.3 log CFU/egg with the HR treatment but not recovered from those treated using HRS (Table 2). *A. niger* spores exhibited the highest tolerance to the hydroxyl-radical treatment although could be decreased by 3 log CFU/egg by applying HRS that was significantly (P<0.011) higher compared to HR (Table 2).

**Table 2:** Log Count Reduction of *E. faecalis, E. coli., P. aeruginosa, Salmonella* and *A. niger* spores inoculated onto ungraded eggs the treated with different hydroxyl-radical treatments. The HR Treatment was when the reactor was operating with 2 % v/v hydrogen peroxide and UV-C dose of 19 mJ/cm^2^ and ozone applied throughout the 10 s treatment time. The Heavy treatment spayed a riboflavin solution on the inoculated egg followed by 3 %v/v hydrogen peroxide at 70 °C prior to passing through the hydroxyl-radical unit.

Microbe	H_2_O_2_ (%)	Riboflavin (mM)	UV-C dose (mJ/cm^2^)	Log CFU/Egg	Log Count Reduction
*Enterococcus faecalis*					
Control				5.40±0.08	
HR^1^	2	0	19	Not Detected	>5.40a
HRS^2^	3	13.75	114	Not Detected	>5.40a
*Escherichia coli*					
Control				5.71±0.04	
HR	2	0	19	Not Detected	>5.71a
HRS	3	13.75	114	Not Detected	>5.71a
*Pseudomonas aeruginosa*					
Control				5.65±0.14	
HR	2	0	19	1.35±1.91	4.30a
HRS	3	13.75	114	Not Detected	5.65b
*Salmonella*					
Control				5.58±0.24	
HR	2	0	19	Not Detected	>5.58a
HRS	3	13.75	114	Not Detected	>5.58a
*Aspergillus niger*					
Control				4.67±0.05	
HR	2	0	19	3.18±0.52	1.49a
HRS	3	13.75	114	1.59±2.25	3.08b

Means for each microbe followed by the same letter are not significantly different (P>0.05).

1 HR: Hydroxyl-radical process: 2 % hydrogen peroxide, 20 ppm ozone, 19 mJ/cm^2^.

2 HRS: Hydroxyl-radical process: 3 % hydrogen peroxide, 20 ppm ozone. 114 mJ/cm^2^.

### Quality metrics of eggs treated with hydroxyl-radical process or formaldehyde fumigation

With the HR treatment there was no significant change in the cuticle layer, Haugh unit or yolk color compared to the non-treated controls (Table 3). However, with both the HRS and formaldehyde treatments there was a significant removal of the cuticle layer as determined using the Cuticle Blue staining. In comparison there was no significant difference observed in either the Haugh Unit or Yolk Color compared to the non-treated control or those undergoing HR treatment. The result would suggest that the egg membranes remained intact while the cuticle layer had been compromised by formaldehyde or HRS.

**Table 3:** Effect of hydroxyl-radical (HR) with inclusion of riboflavin (HRS) on egg quality metrics compared to formaldehyde gas treatment. Hatchery eggs were treated with the hydroxyl-radical treatments or formaldehyde with the change in cuticle layer being measured via staining with the Haugh Unit and yolk color being determined after 35 day storage at 4°C.

Treatment	H_2_O_2_ (%)	Riboflavin (mM)	Cuticle ΔE	Haugh Unit	Yolk Colour
Control			40.1±5.5a	79.6±10.8a	5.00±0.00a
HR^1^	2	0	37.5±1.2a	82.2±2.4a	4.70±0.30a
HRS^2^	3	13.75	25.9±1.1b	65.3±12.5a	6.00±1.00a
Formaldehyde	0	0	21.1±4.6b	71.9±11.0a	5.3±0.90a

Values within columns followed by the same letter are not significantly different (P>0.05)

1 HR: Hydroxyl-radical process: 2 % hydrogen peroxide, 20 ppm ozone, 19 mJ/cm^2^.

2 HRS: Hydroxyl-radical process: 3 % hydrogen peroxide, 20 ppm ozone. 114 mJ/cm^2^.

### Hatchery and grow-out trials

The hatchery trials were performed on non-inoculated eggs that were treated with HR, HRS or formaldehyde fumigation and compared to non-treated controls. Of the 50 eggs in each of the treatment batches, the non-treated control had the highest hatch rate, which was attributed to the higher embryo viability up to the candling stage (Table 4). If it is assumed that those eggs that failed to develop an embryo were infertile then the highest Hatch to Fertility (HOF) was for eggs receiving any one of the disinfection treatments compared to non-treated controls (Table 4). Of the chicks that hatched, there was one case of unhealed navels within each group, with no red-hocks caused by disrupted blood flow being observed. Two chicks derived from the control (non-treated eggs) died within the first 7-days post-hatched with no losses in groups derived from disinfected eggs (Table 4). The cause of the mortality was inconclusive from post-mortem examination.

**Table 4:** Hatching performance of fertile eggs treated with hydroxyl-radical process or formaldehyde.

Treatment	# Eggs	# Viable Embryos	#Chicks Hatched	Hatch Rate	HOF	#Unhealed Navel	#7-Day Mortality
Control	50	40	32	74 %	80 %	7	2
HR^1^	50	33	32	64 %	97 %	2	0
HRS^2^	50	38	34	68 %	89 %	3	0
Formaldehyde	50	35	33	66 %	94 %	4	0

1 HR: Hydroxyl-radical process: 2 % hydrogen peroxide, 20 ppm ozone, 19 mJ/cm^2^.

2 HRS: Hydroxyl-radical process: 3 % hydrogen peroxide, 20 ppm ozone. 114 mJ/cm^2^.

**Fig. 1:**
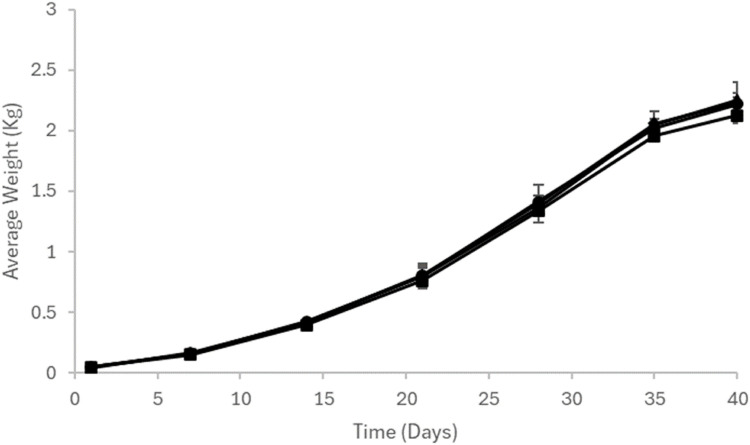
Weight gain of birds derived from eggs treated with hydroxyl-radical process HR (▲) or HRS (•) or formaldehyde (♦) compared to non-treated controls (■). Groups of eggs (n=50) were treated with the egg disinfection method, incubated and hatched. The birds were in groups of five and weighed periodically throughout the 40-day grow-out period.

There were no further chick losses during the grow-out phase and the rate of development of the birds did not differ between those derived from disinfected eggs vs non-treated controls (Fig 1). The feed conversion rate ranged from 1.59 – 1.75 with no significant difference between the different groups (Table 1).

Table 5.

**Table 5:** Feed Conversion Ratio (FCR) of chicks derived from hatchery eggs treated with hydroxyl-radical process or formaldehyde. The groups of eggs (n = 50) were treated with hydroxyl-radical process or formaldehyde then incubated and hatched. The birds were subdivided into groups of 5 birds, and feed intake and weight gain were measured over the 40-day grow-out period.

Group	Average Feed Consumed Per Bird			
	Day 11 Starter Feed	Day 25 Grower Feed	Day 40 Finisher Feed	FCR
Control	0.29±0.02a	1.16±0.06a	1.81±0.10a	1.72±0.16a
HR	0.32±0.02a	1.19±0.03a	2.14±0.11a	1.75±0.22a
HRS	0.31±0.02a	1.17±0.06a	2.01±0.11a	1.61±0.14a
Formaldehyde	0.31±0.02a	1.19±0.06a	1.96±0.10a	1.59±0.07a

Values within columns followed by the same letter are not significantly different (P>0.05).

## Discussion

As previously found, the HR hydroxyl-radical process reported by Zai *et al*. (2023) was sufficient to support a >5 log CFU reduction of *Salmonella*, although it had a negligible impact on the TAC. The reduction of TAC has been previously applied as a metric to assess the effectiveness of eggshell disinfection (Berrang *et al*., 1997; Spickler *et al*., 2011). In the current study, a reduction in TAC could be achieved by increasing the hydrogen peroxide concentration from 2 % v/v to 3 % v/v along with a longer treatment time (i.e. higher UV-C dose and ozone) and inclusion of the photo-sensitizer, riboflavin. It was noted that the hydroxyl-radical treatment (HRS) inactivated the TAC and removed the cuticle layer. The enhancement of the hydroxyl-radical treatment by the inclusion of riboflavin was likely attributed to catalytic action of the vitamin by the propagation of a free-radical reaction that enhanced oxidative action (Chan and Chu, 2009; Sel, *et al*., 2014). Previously, riboflavin has been applied in combination with UV-A light and the current study demonstrated that the photocatalytic activity can be extended to hydroxyl-radicals generated by the action of UV-C. It was noted that the lowest riboflavin concentration supported the highest efficacy with regards to the reduction of total aerobic count. This could be attributed to the possible generation of excess hydroxyl-radicals leading to termination reactions in accordance with the Harber-Weiss reaction (Ab Aziz, *et al*., 2016).

It can be inferred that most of the TAC on the surface of the egg was embedded within the cuticle. An association of the TAC with the cuticle layer integrity has been previously noted in studies assessing the efficacy of egg-washing (Braun, *et al*., 2011; Kulshreshtha, *et al*., 2022).

The cuticle layer is composed of glycoproteins, lipids, polysaccharides, and antimicrobial constituents such as lysozyme, and ovotransferrin, amongst others (Fulton, *et al*., 2012; Rodríguez-Navarro, *et al*., 2013). Despite the apparent inhabitable niche, the cuticle microbiome is considered to be composed of microflora derived from maternal origin and those within the laying environment (Kulshreshtha, *et al*., 2022). The function of the microbiome remains unclear but is considered to prevent attachment and biofilm formation by *Salmonella* via occupying binding sites. In addition, the microflora of the cuticle layer has also been observed to reduce pathogen persistence through competitive exclusion and production of antimicrobials such as hydrogen peroxide (Kulshreshtha, *et al*., 2022). Therefore, the integrity of the cuticle layer is considered to provide both physical and chemical protection against exposure of eggs from microbes within the hatchery environment. (Kulshreshtha, *et al*., 2022).

Despite the HRS hydroxyl-radical process removing the cuticle layer, the inner membrane integrity was preserved, as evidenced by no significant difference in the Hughe units between the control and HR treatment. It was noted that despite most of the cuticle being removed by the HRS treatment, the pores retained plugs that provided membrane protection. It has been previously reported that egg washing removes the surface cuticle layer although cuticle-plugs remain that are sufficient to prevent pathogen invasion and moisture loss (Leleu, *et al*., 2011).

It was found that the HRS treatment could inactivate the test bacteria apart from *P. aeruginosa* that exhibited a higher tolerance to hydroxyl-radicals. It has been reported that *P. aeruginosa* exposed to hydroxyl-radicals induces a protective antioxidant system that enhances cell tolerance (Aharoni, *et al*., 2018). As previously reported, *A. niger* spores exhibited a high tolerance to hydroxyl-radicals due to the protective spore wall (Hasani, *et al*., 2019). Yet, the HRS treatment supported the inactivation of *P. aeruginosa* and reduce *Aspergillus* levels by virtue of the enhanced hydroxyl-radical generation from hydrogen peroxide via the inclusion of riboflavin.

From the hatchery trials, there were no negative effects on embryo development and hatchery rate. The same findings were found by Zai *et al*. (2023) for the HR treatment and, in the current study, the loss of the cuticle layer by HRS and formaldehyde treatments. It has been reported that formaldehyde treatment does not have a negative effect on the hatchery rate when compared to non-treated controls (Kim and Kim, 2010). It was noted that differences in HOF were observed with those in the treatment groups compared to the non-treated controls. HOF assumes that those eggs that failed to develop an embryo were infertile as opposed to fertile eggs that did not progress in development due to the applied treatment. From examining the clear eggs at candling, there was no indication of embryo development. Moreover, considering that no damage to the membranes was observed according to the Haugh units, it can be concluded that the clear eggs were due to infertility. On this basis, the disinfection of hatchery eggs by any of the three tested methods tested provides positive effects on embryo development.

In the current study, there were no negative effects on chick development between the different treatments compared to the non-treated control. The FCR for the chicks during growth was in-line with that typically encountered in broiler production (Keita, *et al*., 2016). Although this may suggest that the HR and HRS do not provide benefits compared to applying no treatment, it should be noted that the pathogen burden could be removed by hydroxyl-radical treatment. It is possible that differences between treatments would more apparent if a less sanitary hatchery was used. This theory can be confirmed by applying treatments with commercial hatcheries.

Two hydroxyl-radical treatments for sanitizing hatchery eggs have been validated and verified. The HR treatment can be applied to inactivate *E. faecalis, E. coli* and *Salmonella* while reducing *P. aeruginosa* and retaining the cuticle layer. The HRS treatment can support the surface sterilization of eggs at the cost of the cuticle layer. Nevertheless, both treatments did not have a negative effect on embryo development or growth performance of the subsequent chicks. Therefore, the hydroxyl radical treatment is a viable alternative to formaldehyde in reducing the carriage of *Salmonella* and avian pathogens associated with hatchery eggs.

## Declaration of interests

The authors can confirm no financial or personal conflict of interest
